# Proteins as Targets in Anti-Schistosomal Drug Discovery and Vaccine Development

**DOI:** 10.3390/vaccines9070762

**Published:** 2021-07-08

**Authors:** Ndibonani Kebonang Qokoyi, Priscilla Masamba, Abidemi Paul Kappo

**Affiliations:** Molecular Biophysics and Structural Biology (MBSB) Group, Department of Biochemistry, Kingsway Campus, University of Johannesburg, Auckland Park 2006, South Africa; nqoks96@gmail.com (N.K.Q.); presh4u@rocketmail.com (P.M.)

**Keywords:** protein–protein interactions, schistosomiasis, praziquantel, S*m*-Tsp-2/Alhydrogel, S*m*-P80/Gla-Se, S*m*14/Gla-Se

## Abstract

Proteins hardly function in isolation; they form complexes with other proteins or molecules to mediate cell signaling and control cellular processes in various organisms. Protein interactions control mechanisms that lead to normal and/or disease states. The use of competitive small molecule inhibitors to disrupt disease-relevant protein–protein interactions (PPIs) holds great promise for the development of new drugs. Schistosome invasion of the human host involves a variety of cross-species protein interactions. The pathogen expresses specific proteins that not only facilitate the breach of physical and biochemical barriers present in skin, but also evade the immune system and digestion of human hemoglobin, allowing for survival in the host for years. However, only a small number of specific protein interactions between the host and parasite have been functionally characterized; thus, in-depth understanding of the molecular mechanisms of these interactions is a key component in the development of new treatment methods. Efforts are now focused on developing a schistosomiasis vaccine, as a proposed better strategy used either alone or in combination with Praziquantel to control and eliminate this disease. This review will highlight protein interactions in schistosomes that can be targeted by specific PPI inhibitors for the design of an alternative treatment to Praziquantel.

## 1. Introduction

Protein–protein interactions (PPIs) have gained interest as potential drug targets for a broad range of diseases, such as tropical infections, cancer and neurological disorders, among others. Interactions between specific pairs or groups of proteins are crucial to all stages of development and homeostasis. As such, several human diseases can be traced to abnormal protein–protein interactions, either through the loss of an essential interaction or through the formation of a protein complex at an inappropriate time or location [1]. The major goal of studying PPIs in disease states is the development of novel therapies targeting interactions that are functionally relevant to disease progression and patient outcomes. Moreover, the identification of disease-specific patterns of PPIs could serve as important disease biomarkers whose selective measurement could in turn lead to improved diagnostics or prognostics for human diseases, including schistosomiasis. Human schistosomiasis is a neglected parasitic disease caused by an infection from digenetic *Schistosoma* trematodes and is one of the greatest threats to public health worldwide, with over 250 million reported cases of infection [2,3]. The disease is endemic in tropical areas and endemicity is dependent on the presence of the intermediate host, an aquatic snail, in freshwater bodies [4]. Estimates suggest annual deaths of over 200,000 individuals worldwide caused by the immunosuppressive and carcinogenic effects posed by infection with this disease [5,6]. In addition, it increases susceptibility to other harmful ailments, including hepatitis B, malaria, bladder cancer and human immunodeficiency virus (HIV) infection, which cause a high disease burden in schistosomiasis-endemic regions [7]. The most clinically important species are *Schistosoma mansoni*, which is the most common species in Africa, transmitted by *Biomphalaria* snails, that causes intestinal and hepatic disease; *S. japonicum*, transmitted by *Oncomelania* snails, which results in intestinal schistosomiasis in China, Indonesia and the Philippines [8]; and *S. haematobium*, another common African species that is transmitted by *Bulinus* snails and causes urogenital schistosomiasis [9].

Schistosomes have complex life cycles involving two hosts: an intermediate snail host and the definitive human host. Their development begins when eggs are released from an infected individual, either through urine or feces, and find their way to fresh water where they hatch and develop into free-swimming miracidia. The miracidium seeks and locates its intermediate host, the snail, where it reproduces and gives rise to multicellular sporocytes. These develop into cercariae (larvae), consisting of two embryonic suckers and a two-branched tail, which are capable of infecting humans [10]. To meet the World Health Organization’s (WHO’s) goal of eradicating the disease by 2025, alternative methods to assist the currently used drug, Praziquantel (PZQ) becomes imperative. For over 20 years, PZQ has become the most effective, common and widely used drug for the treatment of schistosomiasis due to its cost effectiveness, availability and minimal side effects on the patient. It is a pyrazine-isoquinoline derivative, first discovered in 1972, and was primarily developed for veterinary use against cestode infection [11]. It was later employed for the treatment of schistosoma infections, and since then, no therapeutic advancement has been successfully made for an alternative drug against schistosomiasis [12]. PZQ is a white crystalline powder, bitter to taste, melts at 136 to 140 °C and is practically insoluble in water but is highly soluble in organic solvents such as chloroform and dimethylsulfoxide [13]. It is a combination of two stereoisomers, one responsible for the anti-schistosomal properties the drug possesses, while the other contributes to the minimal side effects presented by the drug and the bitter taste of the drug, making it quite difficult for small children to swallow [14]. PZQ is usually supplied as four-side shaped tablets containing 600 mg of the active ingredient, and the recommended dose is 40–60 mg/kg body weight [15]. It has been reported that its bioavailability increases with simultaneous administration of food. About 10–60% of treated patients experience mild side effects such as dizziness, nausea, headache and sometimes vomiting. However, even in the advent of the side effects, Praziquantel has been used for mass treatment campaigns particularly targeted at school aged children, who are the most infected group of the population, due to its effectiveness against all schistosoma species infecting humans. The efficacy of PZQ is generally high, with cure rates ranging from 60% to 95%; however, with PZQ being the only anti-schistosomal drug in use for more than three decades, prospects of possible drug resistance are quite high, and there have been reports of PZQ-resistant schistosomes [14,16]. Irrespective of the cases of drug resistance [17], there are other principal drawbacks associated with PZQ: it fails to destroy the worm 2–4 weeks post infection [18], it does not prevent against re-infection [11] and above all, its mechanism of action is still not yet understood [7,12]. Disruption of the lifecycle of the parasite in its infective stages is one approach that can help ease the gradual rise in *Schistosoma* infections [6,19]. The development of drugs and vaccines against schistosomiasis has been delayed over the years by a lack of understanding of the parasite’s survival mechanisms. Schistosomes have a complex six-stage life cycle comprising several morphological stages, occurring in two distinct hosts. In this regard, schistosomes display a great capacity to survive different environmental conditions and an ability to escape the immunological responses of their respective hosts [20]. They achieve this through molecular interactions that favor their survival [21].

### 1.1. Protein Composition of Schistosome Life-Cycle Stages

#### 1.1.1. Eggs

Adult worms produce eggs that are capable of restarting the infection cycle of schistosomiasis. The proteome of schistosome eggs is composed of proteins that play a defensive role in oxidative stress, such as thioredoxin and GST. In addition, triose phosphate isomerase implicated in glycolysis and energy production has been shown to be highly expressed in schistosome eggs [22]. Sm40 is reportedly in high abundance in eggs and is known to play an inhibitory role in granuloma formation by minimizing potential fibrosis and cell proliferation induced by other schistosome egg proteins [23]. Additionally, calmodulin is expressed in abundance at this stage and is reported to be an essential protein for egg development. In the same vein, a polymorphic mucin variant is secreted by eggs in order to escape host immune responses by acting as a ‘smoke-screen’, blocking the recognition of eggs by receptors of the host defense mechanisms [22,24]. The alpha-1 glycoprotein is located in the subshell area of mature eggs, where it regulates IL-4-mediated Th2 responses, and is reported to induce a potent anti-inflammatory response by inducing regulatory B cells to produce IL-10 [25].

#### 1.1.2. Miracidia

A highly expressed in the miracidia of *S. mansoni* is the *S*. *mansoni* protein 40 (Smp-40), which is reported to contribute about 15% of the soluble proteins of miracidia [26]. This protein has been studied for its role in blastogenic reactions, granuloma responses and as an immunodiagnostic target for schistosomiasis [23]. Ludolf and colleagues [27] reported that Smp-40 can be used as a potential antigen for schistosomal immunodiagnostic tests following its effective immunoprecipitation by *S. mansoni* chronically infected mouse serum. In addition, this protein is a member of the small heat shock protein (sHSP) family; it elicits its chaperoning function by assisting Hsp60 in capturing unfolded proteins that form stable complexes, thereby resulting in efficient disaggregation of the protein aggregates [28]. The miracidium faces a number of unfavorable physiological stresses after hatching and locating the snail host. For this reason, other members of the sHSP family upregulated at this stage include Hsp20, Hsp16, Hsp27 and Hsp40. The abundance of sHSPs is believed to provide the miracidium with protection against stress, while ensuring that glucose reserves that provide energy to the miracidia are not overused [29].

Another protein found to be highly expressed in the miracidium stage of the parasite is a 78 kDa glucose-regulated protein (GRP78), which is a member of the heat shock protein 70 (HSP70) family. GRP78 plays a role in the folding of unfolded and partially folded proteins within the endoplasmic reticulum and is involved in the transport of secretory proteins. It is, therefore, proposed to function in a similar manner in stabilizing the macromolecular structure for miracidium proteins destined for secretion [30]. Mitochondrial import receptors are reported to be expressed in abundance in the miracidial stage and their expression may be attributed to the high energy requirement for miracidium growth, development and host-seeking activity within the first 5–6 h after hatching. Identified mitochondrial import receptors in the miracidia are TOM34 and TOM40, responsible for translocation of proteins into the mitochondria [29]. Cathepsins D and L have been identified in the proteome of miracidia; these proteases play a role as digestive enzymes in parasitic helminths [31]. The aspartic protease cathepsin D has been found in the schistosome gut as an apical enzyme that plays a significant role in the digestion of hemoglobin released from ingested erythrocytes. These two proteases might play specific roles in miracidia metabolism, including regulating digestion of the snail’s hemoglobin after infection [29,32].

#### 1.1.3. Cercariae

A number of proteins are expressed in the infective stage of schistosomes, which include a group of proteins involved in calcium binding, calcium regulation and calcium-activated functions. Two of these proteins paramyosin and SPO-1 have been shown to play a role in immune evasion, while elastases are implicated in the degradation of host skin barriers. Cercarial elastase (SmCE) is the major invasive larval protease in schistosomes, secreted by the cercariae to aid mammalian host skin invasion by breaking various skin barriers [33]. An elucidation of SmCE in facilitating host penetration is exhaustively discussed later in the review. Moreover, a tegument-bound antigen, Sm14, which forms part of the fatty acid binding proteins, is found to be highly expressed during this stage of schistosomal development with critical roles in the uptake as well as transport of fatty acids from the host. This has also been detailed in later sections of this review. Other proteins that are abundant in the cercariae are soluble glycolytic proteins such as triose-phosphate isomerase, GAPDH and phosphoglycerate kinase, which are known to provide the energy required by the cercariae for mobility while locating a human host [34].

#### 1.1.4. Schistosomula

Upon entry of the cercariae into its definitive human host, it transforms into a schistosomula which then matures into an adult worm. This stage is characterized by a drastic downregulation of the SmCE and upregulation of heat shock proteins (Hsp16, Hsp60 and Hsp70). The upregulation of HSPs in the earliest stages of intra-mammalian schistosomula development is suggested to be in response to the extensive change in niche environments, which include substantial thermal changes between freshwater and the human body [35,36]. A member of the tetraspanins family, S*m*-TSP-2 is reported to be expressed at this stage and is responsible for tegument formation [37]. The status of this protein in vaccine development is extensively reviewed within a later section of this manuscript.

#### 1.1.5. Adult Worms

Adult worms have the ability to move through different tissues within the human body, such as the liver and lungs. The fatty acid binding protein, Sm14 [38], chaperones such as Hsp60 and Hsp70 and structural proteins such as actin, tubulin, titin, paramyosin and myophilin are found to be expressed in abundance during this stage of the parasite [39]. Moreover, several cytosolic glycolytic enzymes such as enolase, pyruvate kinase and GAPDH have been found to aid in energy production in adult worms [40].

## 2. Protease Inhibitors and Schistosomiasis

Proteases are crucial for the survival of parasites, including schistosomes. A number of different proteolytic enzymes play a critical role in facilitating host invasion, nutrient uptake, hatching, evasion of the immune system and modulation of the host physiology [41]. Schistosome research has looked at cysteine (thiol) and aspartic (pepsin, cathepsin, rennin) proteases that schistosomes use as part of their survival tactics, but most attention is directed at serine proteases (trypsin/chymotrypsin-like). The most extensively studied serine protease in schistosomes is the cercarial elastase (SmCE), which is largely responsible for skin invasion by infective schistosome larvae [42]. Protease inhibitors play an important role in restraining potentially detrimental excess proteolytic activity by proteases. In accordance with the type of protease enzyme they inhibit, protease inhibitors are grouped into serine protease inhibitors (serpins, Kunitz-type, Kazal-type), cysteine protease inhibitors (cystatins), metalloproteinase inhibitors and alpha-2-macroglobulin. These inhibitors bind to the substrate to perform their function in various ways and are classified into four major categories: (i) those that block the active site of the protease (canonical inhibitors) [43]; (ii) those that bind a region adjacent to the binding site (exosite inhibitors); (3) those that bind the enzyme through a combination of canonical and exosite-binding mechanisms; (4) allosteric inhibitors that bind the enzyme in any place other than the active site [44].

### 2.1. Serpins

Proteomic analysis of *S. mansoni* larval secretions revealed a number of parasitic proteins that could potentially play a role in host immune evasion and/or promotion of parasite survival. A superfamily of macromolecular serine protease inhibitors (serpins) is one class of proteins that is proposed to function in the pathogenesis of schistosomiasis. Serpins are an ancient class of structurally conserved macromolecular inhibitors of cysteine and serine proteases that are found in nearly all biological systems, including plants, viruses, insects, prokaryotes and animals, such as members of the genus *Schistosoma*. In mammals, serpins play a critical role in the regulation of proteases that control blood coagulation, fibrinolytic pathways, apoptosis and inflammation [45]. All serpins are 330–500 amino acids long, with a molecular weight ranging from 40–60 kDa. Their structure characteristically contains three β-sheets (A, B and C), eight or nine α-helices and a single reactive central loop (RCL). The inhibitory activity of serpins is centered on the RCL [46]. In the native form, it lies outside the main body of the serpin, between β-sheets A and C, and serves as the protease ‘bait.’ The RCL is composed of approximately 20 amino acid residues (P17-P4) with the P1 side chain fitting into the S1 specificity pocket of the target protease, with cleavage of the RCL occurring at the P1-P1′ position. The reactive site (designated P1 position) in the inhibitory loop is determinant and controls much of the inhibitory activity towards the specific targeted protease. It has been proven that the presence of either Lysine or Arginine at the P1 position obstructs any protease from cleaving adjacently to those residues in the protein substrate, while the Asn13, Tyr17 and Tyr18 stabilize the canonical loop in the reactive site [47]. These features are central to the biological activity of the protein and permit the orientation of its functional moieties [48]. At the outset of interaction, the serpin binds to proteases through a non-covalent Michaelis-like complex, which is formed through interactions with residues flanking the scissile bond (P1-P1′) within the RCL, forming a transient covalent ester linkage [49,50]. Following proteolytic cleavage, the RCL is inserted into the body of the serpin, completing the antiparallel organization of β-sheet A. The crystal structure of two trypsin (protease)-α1-antitrypsin (serpin) reveals that once the complex forms between the protease and the serpin, translocation distorts the structure of the protease by 37%, thereby reducing its ability to perform its function, while the serpin itself remains largely unchanged [51].

### 2.2. Schistosome Serpins

The function of serpins in schistosomes remains speculative, although a considerable number of serpins have been identified in the three *Schistosoma* species known to infect humans [34]. The phylogeny of various serpins in schistosomes has been evaluated by searching parasite databases for proteins homologous to the canonical serpin α1-antitrypsin, and eight complete serpin sequences have been identified in the *S. mansoni* gene database, three in the *S. japonicum* database with one partial sequence and one in the *S. haematobium* database (Table 1). With the exception of Sjp_0113720, alignment of the *Schistosoma* serpins with α1-antitrypsin and superimposition of the structural components of α1-antitrypsin indicate that these proteins contain all elements necessary for functionality [50], although their function within the worm has not been reported on. The amino acid sequence of ShSPI, Smp_090080 and Smp_090090 was reported to consist of an unusual feature for inhibitory serpins. Within the RCL of most inhibitory serpins, the P12-P9 region is characterized by amino acids with short side-chains (Ala, Gly, Ser) [52]; however, this feature is not present in these genes and this may signify a non-inhibitory function. Although most genes show over 50% similarity to known mammalian serpins, very little research has been reported in the literature on the identified serpins so far.

### 2.3. Functionally Characterized Schistosome Serpins

#### 2.3.1. Elafin

Skin invasion is the initial step of infection by schistosomes when the freshwater-dwelling cercariae penetrate the skin of the human host. The human skin is a formidable barrier consisting of structural proteins in the epidermis, basement membrane and extracellular matrix, which form a mechanical barrier [58]. To break this obstruction, the schistosome has to degrade the protein matrices and have the ability to minimize the immune response of the skin. Upon contact with the human host, successful cercarial penetration can be achieved within 1.5 min, with the assistance of SmCE [4,59]. The enzyme SmCE is responsible for the degradation of skin macromolecules, including elastin, chondromucoprotein, keratin, fibronectin, laminin and collagen IV and VIII [60], to allow for the penetration of cercariae, which when inside the human host develop into mobile schistosomulae [42,61]. A study by Becker and co-workers reported that a serine protease inhibitor, elafin, may be a novel substrate for SmCE; it would inhibit the action of SmCE during the cercarial stage of the parasite and act as an effective barrier against infection by preventing successful penetration of the cercariae [5]. Elafin is a low molecular weight human protein that is produced naturally in the skin, lung and breast, protecting these tissues from destruction by the immune system [62]. This protein plays an important role in wound healing in the dermal immune response in humans, while also acting as an antimicrobial agent against other pathogens such as *Pseudomonas aeruginosa* and *Staphylococcus aureus*. Elafin is known to bind with high affinity to both human leukocyte elastase and porcine pancreatic elastase [63]; hence, a similar pattern on the level of affinity binding to SmCE was proposed [64]. An earlier study proposed that increased concentrations of elafin may be sufficient to act as an effective barrier against host penetration by the cercariae [65]. A similar study confirmed that applying a serine protease inhibitor to the skin prior to exposure to cercariae prevents its invasion [66]. These studies, in conjunction with the proposed role of elafin in schistosomiasis, suggest that elafin could contribute significantly to schistosomiasis-associated treatment strategies. The ability of elafin to block invasive enzymes makes it an interesting target in drug development. Its proposed interaction with the major destructive serine protease of schistosomes presents an opportunity for intense experimental validation that may provide insights into the development of a drug that can potentially prevent the onset of schistosomal infection.

#### 2.3.2. SmPi56

Immunity to schistosome infection has been assigned to several immune mechanisms, including parasite opsonization by specific antibodies and a number of different effector cells, including neutrophils, macrophages and eosinophils. The mechanisms by which effector cells cause the death of the parasite are not well understood; however, a number of potentially toxic compounds are released by effector cells after reaching the surface of a schistosomulum and these are most likely intended to destroy the parasite. Neutrophils are reported as the first cell type of immune cells to arrive at inflammatory sites [67]. These cells play a critical role in innate immune response development in addition to their involvement in host tissue damage through secretion of proteases and cytotoxic mediators [68]. Neutrophil elastase (NE) is a critical protease in the immune response belonging to the serine protease family, which plays an important role in host defense mechanisms in both physiological and disease-associated conditions [69]. It has been reported that schistosomula and adult worms are sensitive to both pancreatic elastase and NE. To counteract the toxic effects of these proteases, schistosomes produce protease inhibitors, the best known so far being a serpin, Smpi56 [70,71]. In schistosomes, serpins have been postulated to control the homeostasis of serine proteases in the parasites themselves, as well as in their mammalian hosts [57]. SmPi56 (*Schistosoma mansoni* protease inhibitor, 56 kDa) was the first schistosome serpin to be functionally characterized from crude extracts of *S. mansoni* adult worms and was postulated to protect the schistosome from potentially damaging proteases such as NEs that are produced by the host during schistosomulae migration through various tissues in the human body [72]. This protease inhibitor is located in the tegument of adult male and female worms [57] and was found to be capable of specifically binding to pancreatic and neutrophil elastases, thereby protecting the schistosome from activated neutrophils during inflammation that could potentially kill the parasite [50,73]. For this reason, SmPi56-specific inhibitors that could target and block its protective role in adult worms present an interesting approach in anti-schistosomal drug development.

#### 2.3.3. SmSrpQ

At multiple life cycle stages of the schistosome, the parasite moves through host tissues and breaches substantial structural barriers, including the extracellular matrix. The process of early human infection is well-characterized at the multi-cellular larval stage, where the cercariae directly penetrate host skin in a process that is largely facilitated by various secretions from a glandular network that runs the length of the larval body. These secretions contain several histolytic proteases that assist in skin penetration [66]. The larvae of *S. mansoni* invade their mammalian host with the assistance of a serine protease, SmCE, to degrade macromolecular proteins in host skin. The catalytic activity of serine and cysteine proteases after activation is largely regulated by serpins. SmSrpQ, one of two *S. mansoni* serpins found in larval secretions, is only expressed during larval development and in the early stages of mammalian infection, and is responsible for regulating the activity of the major acetabular gland secretions released by skin-invading cercariae to aid in penetration [74]. It is proposed that SmSrpQ aids in protecting the parasite from its own elastase, while its tight regulation of the enzyme is also beneficial to the mammalian host, as it helps to limit host tissue damage at the site of invasion. SmSrpQ is expressed from the onset of development in daughter sporocysts until the one-day-old schistosomula stage and is not detected in either adult worms or the egg/miracidium stage. Moreover, localization of SmSrpQ to SmCE after skin penetration suggests it is unlikely that SmSrpQ interacts with SmCE within the parasite. SmSrpQ is localized in the head gland, post acetabular and a disparate set of pre-acetabular glands, whereas SmCE is found only in the adjacent set of pre-acetabular glands [74]. This suggests that SmSrpQ only plays a regulatory role in the early stages of infection when the cercariae invade host skin and enter dermal blood vessels. In addition, SmSrpQ does not interact with mammalian host proteases such as NE. Inhibition of the interaction between SmSrpQ and SmCE could alter the regulation of elastase production by the worm, which may have detrimental effects on the parasite itself, thereby preventing successful host penetration. Therefore, inhibitors of this protein complex may play a role in studies targeting the infective stage of schistosomes.

Z-AAPF-CMK is an effective inhibitor of cercarial invasion and a known inhibitor of SmCE [75]. It is reported that the presence of this inhibitor results in a significant reduction in complex formation of the SmSrpQ to SmCE complex [74,76]. This suggests that a complex between the two proteins is only formed in the presence of active SmCE, making Z-AAPF-CMK an interesting target for breaking the interaction between the two proteins, and hence, reducing the chances of protection from uncontrolled elastase production by the worm, which may potentially cause harm to the schistosome itself and potentially prevent successful transmission of the disease.

#### 2.3.4. *Sm*KI-1

Schistosomes are able to survive for prolonged periods in the blood system, in spite of continuous contact with coagulatory factors and mediators of the host immune system. Protease inhibitors play a critical role in host immune modulation, in so doing promoting parasite survival under extremely hostile environments. The Kunitz-type serine protease inhibitors are part of the serine protease inhibitor superfamily found in almost all organisms. They are generally small proteins consisting of around 60 amino acid residues and are characterized by one or more Kunitz motifs: α + β with two β strands and two short α helices at the end of the domain [77]. These inhibitors have been shown to have significant physiological functions in a number of organisms. In invertebrates, Kunitz inhibitors have been known to be involved in various physiological processes, including blood coagulation, fibrinolysis, inflammation and ion channel blocking [78]. However, these proteins are not well studied in parasitic helminths and only two have been functionally characterized in schistosomes. The *S*. *mansoni* Kunitz-type protease inhibitor (*Sm*KI-1) is found in the tegument of adult worms and the sub-shell region of eggs. SmKI-1 has been shown to interact with and inhibit several proteases, including NE, which is critical in triggering immune response reactions. Morais and co-workers [67] conducted a study to evaluate the anti-inflammatory potential and the ability of *Sm*KI-1 to interfere with neutrophil migration and function in mice infected with schistosomiasis. Treated mice developed liver damage with a significant reduction in both neutrophil accumulation and elastase activity, confirming the proposed inhibitory effect *Sm*KI-1 has on NE. In addition, *Sm*KI-1 inhibits trypsin and chymotrypsin, which are serine protease digestive enzymes responsible for the degradation proteins in the small intestine. The secretion of *Sm*KI-1 from schistosome eggs implies that it has an additional function of providing protection from these proteolytic enzymes in the gut, as many eggs pass through the intestinal wall and traverse into the gut lumen prior to being excreted in human feces to the external environment [71]. In the mesenteric veins, schistosomes release eggs into the blood, where they are exposed to host attacks, triggering the production of SmKI-1 to confer protection from the immune system [79]. The outer surface of the adult schistosome tegument consist of a double membrane structure, which plays an essential role in modulating host responses as well as ensuring parasite survival. As a protein localized in the tegument, it is suggested that SmKI-1 is involved in offering protection to the parasite in its mammalian host [80].

The adult worm pairs in the mesenteric veins of the small intestine of the host’s blood vessels leads to hypercoagulability through changes in blood flow and endothelial function [81]. This feature and the negatively charged surface of schistosomes is most likely to trigger prompt activation of molecules involved in initiating blood coagulation. Factor XII in blood becomes activated following exposure to a negatively charged surface, such as that presented by *S. mansoni*, thereby triggering the intrinsic coagulation pathway. A cascade of reactions then occurs, leading to the activation of the common coagulation pathway, which ultimately results in the formation of a fibrin clot [82]. SmKI-1 has been reported to inhibit the proteolytic activities of coagulation factor Xa, thrombokinase and plasma kallikrein, a serine protease involved in blood coagulation. This inhibition, as well as the delay in prothrombin time (PT) and activated partial thromboplastin time (APTT) for blood clot formation, suggests possible roles of SmKI-1 in both anti-inflammatory and anti-coagulation processes in the host [71]. Its potential as a vaccine candidate is discussed later in the review.

#### 2.3.5. SjKI-1

According to Virchow’s triad, an alteration to normal blood flow, damaged endothelium or hypercoagulability of plasma initiates blood coagulation [83]. Adult schistosome worm pairs present in the blood vessels cause blood turbulence and disturb endothelial cell functions, thereby activating platelet formation and ultimately blood coagulation. As previously stated, the highly negatively charged nature of the schistosome surface also contributes to the activation of platelets and the intrinsic coagulation cascade [81]. In contrast to *S. mansoni* female worms, which individually deposit hundreds of eggs on a daily basis, those of *S. japonicum* deposit thousands of eggs daily into the mesenteric veins of the mammalian host [84]. Although these eggs are large enough to disturb blood flow in the smaller veins, clotting does not occur [71,85]. This suggests that schistosomes must have evolved mechanisms to control host hemostasis, while also inhibiting blood coagulation to ensure their long-term survival in the host blood stream.

In line with the proposed involvement of schistosomes in blood coagulation, a serine protease inhibitor belonging to the Kunitz type of serine proteases, SjKI-1 has been identified in S. *japonicum* species. SjKI-1 is localized in adult worms and eggs with very low expression in cercariae and the schistosomula [71]. The activity of the intrinsic and common coagulation cascades is measured by APTT and can be prolonged by deficiencies in factors XII, XI, IX, VIII or pre-kallikrein [86]. It has been reported that in the presence of SjKI-1 there is prolonged APTT, suggesting that this Kunitz protein can inhibit one or more clotting factors involved in the coagulation pathways [71]. A vast majority of blood coagulation pathways are calcium-dependent; hence, it is well known that calcium is a critical component in coagulation pathways. SjKI-1 is reported to be able to bind calcium ion successfully; this binding is postulated to contribute to the disruption of the coagulation cascade by reducing the level of free calcium ions available for the coagulation reactions [87], in this way allowing for prolonged survival of schistosomes in the blood vessels. Furthermore, SjKI-1 binds and forms a complex with both trypsin and NE, inhibiting their immune response activity with an IC_50_ value of 0.21 nM. SjKI-1 does not contain a signal sequence [71] and this suggests that it is not secreted via the conventional process involving cleavage of the signal peptide, but is purposely released by the parasite through an unknown excretory mechanism to facilitate its survival. This suggests the likelihood of the involvement of this protein in various biological roles promoting the survival of the schistosome parasite through its interaction with serine proteases. SjKI-1 should be evaluated further as a potential anti-coagulant as well as a potential drug target for schistosomiasis and other hematological disorders.

#### 2.3.6. SjB10

It has been proposed that parasite-derived protease inhibitors, such as serpins, play essential roles in the survival of parasites through host immune evasion by interfering with hosts’ immunomodulatory signals, as well as protecting the parasite from the damaging effect of exogenous host proteases [72]. In addition, serpins participate in the maintenance of cellular homeostasis through extensive regulation of endogenous proteases [88]. To date, a number of serpins have been isolated and recombinantly produced from a range of parasitic helminths including *S. mansoni* [89], but there are limited studies on the identification and characterization of serpins from *S. japonicum and S. haematobium*. These include a 64 kDa serpin from *S. japonicum* SjB10, which was identified and reported to be localized in the cercariae, schistosomulae, eggs and adult male worms. This protein has an overall low sequence homology (21–65% homology) with serpins from other parasitic helminths; however, the presence of special characteristic features in its structure, such as the serpin motif and RCL, identify this protein as a serpin [90]. Moreover, its molecular weight of 64 kDa is consistent with other members of the serpin superfamily [51]. In addition, secondary and tertiary structure prediction analysis show that SjB10 consists of 9 α-helices and 15 β-strands, correlating with other known serpins [51]. The predicted tertiary structure of SjB10 has exposed RCLs; this special feature of inhibitory serpins makes them easily accessible to target proteases [91]. These special characteristics support the notion that SjB10 is a member of the serine protease inhibitor superfamily.

**Figure 1:**
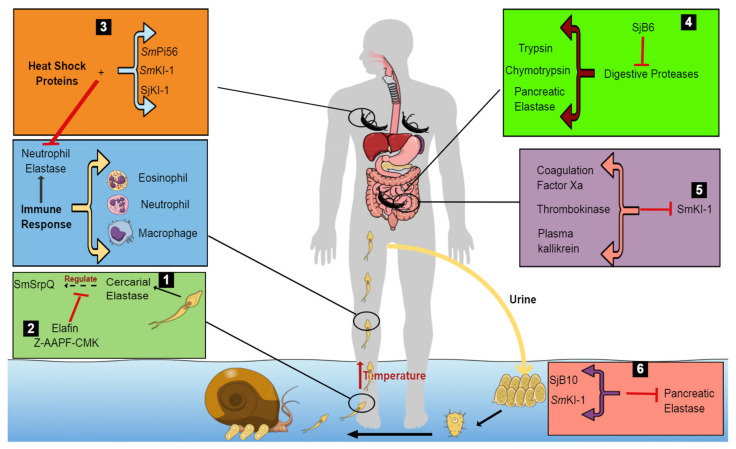
Model proposing the survival mechanisms of schistosomes within the host by protease inhibitors. The cercariae penetrate the human skin using cercarial elastase, which secretes a protease inhibitor, SmSrpQ. This regulates the production of cercarial elastase, in this way protecting the cercariae from its own elastase (1). The presence of CE inhibitors, Elafin and Z-AAPF-CMK, inactivates CE, blocking SmSrpQ regulation, and thus, preventing successful host penetration (2). However, in their absence, the cercariae migrate through various tissues in the host. This stimulates an immune response in the human host, leading to the release of effector cells and specific proteases such as NE to defend the human body (2). To counteract the host’s immune response, schistosomules secrete various protease inhibitors (SmPi56, SmkI-1 and SjKI-1) and heat shock proteins to inhibit the effects of NE (3). In the small intestine (4), schistosomes escape digestion by digestive enzymes through the secretion of SjB6, which inhibits digestive proteases within the host (trypsin, chymotrypsin and pancreatic elastase). In the mesenteric veins of the host, adult worms prevent blood coagulation by secreting SmKI-1, which inhibits various blood-clotting factors, in this way interfering with blood flow to vital organs of the body (5). The worms survive and produce eggs to continue the infection cycle. These eggs are protected from the effects of pancreatic elastase by SjB10 and SmKI-1 (6).

The amino acid sequence within the RCL of a serpin is a critical determinant of not only its specificity to target proteases, but also whether the serpin is inhibitory or non-inhibitory [92]. This is dependent on the consensus 20/21 residue peptide ‘P17 [E]-P16 [E/K/R]-P15 [G]- P14 [T/S]-P13 [X]-P12−9 [AGS]-P8−1 [X]-P1′–4′’ [90]. For inhibitory serpins, P15 is usually a glycine residue, P14 is a threonine or serine and positions P12–P9 are usually occupied by short side-chain residues, such as alanine, glycine or serine. The short side chain residues allow easy and efficient insertion of the RCL into the ‘A’ β-sheet, a process critical to protease inhibition and subsequent conformational change in the serpin molecule. In contrast, the corresponding regions of non-inhibitory serpins do not conform to this consensus sequence [90]. SjB10 showed high conservation of primary, secondary and tertiary structure in correlation to known serpin inhibitors, suggesting that this protein most likely has an inhibitory activity. This is also supported by the high accumulation of hydrophobic amino acid residues in its hinge region, which is thought to be useful for the construction of the skeleton conformation necessary for the inhibitory activity of serpins [93]. The function of SjB10 is still speculative; however, owing to its expression in the cercarial stage, it may be released during host skin penetration to facilitate the inhibition of the host’s proteases. Several proteases of the mammalian host have been tested in vitro and SjB10 is said to exhibit an inhibitory effect only on pancreatic elastase. The localization of SjB10 in the anterior gut of the adult worm and von Lichtenberg’s layer of the egg [94] proposes a possible protective role of SjB10 for the schistosome against digestion by host proteases. The *S. japonicum* egg shell has an electron opaque matrix with two layers interrupted by preformed pores [95]. Schistosome eggs must migrate through tissues of the intestine or bladder, leaving the host to re-start the life cycle, and this is facilitated by the secretion of special immune-reactive molecules from the outer envelope surrounding the differentiating miracidium [95,96]. During the release of secreted antigens into the surrounding host tissue through the preformed pores on the egg shell, host molecules, such as immune and digestive proteases, could consequently gain access to the egg through these pores. The localization of SjB10 within the von Lichtenberg’s layer of the egg suggests a possible protective role by this protein for the parasite against a potential proteolytic attack by host digestive and immune proteases present within the intestine as the eggs migrate through this tissue [97]. SjB10 presents an interesting potential target in schistosome drug development through targeting SjB10-specific protein inhibitors. The various protease inhibitors that have critical functions in the survival and development of schistosome worms are shown in Table 2 and their functions are summarized in Figure 1.

## 3. *Sm*29 Protein

Currently, schistosomiasis control strategies are mainly focused on the treatment of infected individuals by chemotherapy with the safe and effective drug PZQ [99]. Despite decades of chemotherapy, the number of infected people is still almost the same, especially in resource-poor settings with little or no access to proper health care facilities [100]. Constant re-infection of individuals, together with poor sanitary conditions in tropical countries, necessitates venturing into control strategies other than dependence on a single drug treatment plan [100]. Vaccination could be an interesting approach to supplement the available drug in an attempt to reduce infection rates. In schistosomiasis a vaccine that results in even a partial reduction in worm burdens could potentially reduce pathology to a considerable level and limit parasite transmission [101]. After decades of repetitive treatment with the preferred drug for schistosomiasis, many world health agencies support the notion that immunization with an anti-schistosomal vaccine presents a better long-term strategy to control schistosomiasis [102]. Hence, there has been an increase in studies looking into testing different antigens of the parasite and various vaccination strategies [101]. Proteins that are secreted or fixed on the surface of schistosomes are easily exposed to host tissues, and thus, have a higher chance of presenting as potential candidate molecules for the development of new vaccines. In mice, it has been reported that isolated tegumental membrane antigens can stimulate protective immunity [103]. Studying proteins anchored in the tegument of schistosome worms is, therefore, essential to improve functional understanding of the tegument at a more advanced level to identify molecules that may act as targets for protective immune responses or may be useful in diagnosing schistosomiasis [104].

*Schistosoma mansoni* 29 kilodalton protein (*Sm*29) is a membrane-bound protein that is highly expressed on the tegument of *S. mansoni* worms; its expression is not seen in the internal tissues of the parasite. The amino acid sequence of *Sm*29 possesses a signal peptide and a transmembrane region as well as cysteine-rich domains that form disulfide bridges, which confers stability to this glycoprotein [105]. This transmembrane protein is present in the schistosomula and adult stages of the parasite, and has been implicated in several immune response interactions, thus making it an important vaccine target.

Previous analyses of immune responses in experimental and human schistosomiasis have shown that antibodies are capable of killing schistosomula in the presence of phagocytic cells, such as macrophages, platelets and eosinophils, and these have been reported to be able to confer resistance to infection [106]. Thus, studying the different functional properties of antibody isotypes is essential to understand the predictive information regarding disease progression and the efficacy of vaccination. Extracellular pathogens enter the human body across the epithelial barriers of the mucosa lining the respiratory, digestive and urogenital tracts, or through damaged skin, and can then establish infections in the tissues. Antibodies must, then, be widely distributed to neutralize or completely eliminate the pathogen. A study was conducted to investigate the antibody isotype profile specific to Sm29 antigen in the sera of resistant individuals in comparison to ones who are susceptible to infection and re-infection in a schistosomiasis-endemic area in Brazil. Two Sm29-specific antibodies, IgG1 and IgG3, were used in conducting this investigation and it was reported that isotypes related to opsonization, cell-dependent cytotoxicity and activation of the classical complement pathway were present in significant levels in individuals resistant to infection and re-infection [105]. This, therefore, presents the Sm29 antigen as a target to antibody-dependent cell cytotoxicity (ADCC) effector mechanisms mediated by IgG1 and IgG3. Furthermore, these antibodies, in addition to their role in opsonization, cell-dependent cytotoxicity, and the ability to activate the classical complement pathway, are actively involved in protective immunity of the host [107]. IgG2 and IgG4 are often released in response to antigens activated during *S. mansoni* infection to block the effect of protective antibodies mediating ADCC reactions against the schistosomula by reacting with parasite surface glycoproteins, preventing the functional effector mechanisms involved in parasite killing. In the presence of Sm29, significantly low levels of these antibodies are observed, implying that this antigen does not stimulate the production of these antibodies [108]. The IgG4 response has also been linked to increased susceptibility to infection [109]. It can, therefore, be concluded that the absence of IgG2 and IgG4 in the presence of this transmembrane antigen might be an important humoral attribute that contributes to the protection of the mammalian host against schistosomiasis and presents S*m*29 as a potential vaccine candidate.

It has also been reported that mice immunized with S*m*29 showed high levels of protection with significant reduction in worm burden, liver granulomas and intestinal eggs. This protection is associated with a typical Th1 immune response. In addition, schistosomes that were recovered from S*m*29-vaccinated mice showed no upregulation of heat shock or chaperone genes that are usually employed by these parasites to escape unfavorable developmental stages [105]. S*m*29 is reported to form part of the 16% of highly expressed proteins in *S. mansoni,* suggesting that it plays an important role in the biology of the worm [110]. It has been reported that one of the proteins found to interact with *Sm*29 is an inhibitor of the membrane attack complex, CD59, which is mainly used by many viruses as an immune evasion tactic [5]. Experimental exploration of the interaction between *Sm*29 and CD59 could provide insights on how *S. mansoni* parasites inhibit the membrane attack complex and give rise to strategies that could prevent this inhibition.

## 4. *Sm*14 Protein

Schistosomes are known to lack the oxygen-dependent pathways required for the synthesis of sterols and fatty acids. For this reason, these parasites are solely dependent on their host for these and other essential lipid supplies [111]. Schistosomes are only capable of synthesizing phospholipids and triacylglycerols from precursors obtained from the host, such as the low-density lipoproteins of the blood in which the parasites reside [112]. These parasites have a strong requirement for lipids for the synthesis and maintenance of their complex membrane systems. Fatty acids act as precursors for lipid and phospholipid synthesis and it is, therefore, safe to imply that they are a critical component in the life cycle of the parasite, playing a role in membrane formation, functioning as lipid anchors for proteins and sexual maturation and regulating egg production [38,113]. Fatty acid-binding proteins (FABPs) are predominantly important for the uptake, transport and compartmentalization of host-derived fatty acids and are considered a potential target for vaccine development. The 14 kDa *S. mansoni* (Sm14) antigen was the first FABP homologue identified in helminths [38,114]. Sm14 elicits a T helper cell type 1-(Th1) mediated immune response that is associated with resistance to schistosomal infection in individuals from endemic regions in Brazil [38]. This correlates to an earlier study that proposed that this protein stimulates a protective response against schistosomiasis [115]. Sm14 has been postulated to interact with Sm29 and the fusion protein is termed FS*m*14/29 [116]. Taking into account the critical functions of both antigens in schistosomal infection, their combined effect has been tested and will be explained later in this review.

## 5. Heat Shock Proteins

For schistosomes to survive, their proteome needs to be able to meet the demands of the hostile conditions associated with the alternating hosts and environments characterized by variable physiological conditions such as changes in pH, temperature and nutrient supply [117]. In contrast to free-living parasites, schistosomes are prone to undergo extensive molecular evolution during their developmental cycles, promoting the chances of producing mutated proteins and making their proteome aberrant. It is for this reason that schistosomes require a robust protein folding system to increase their survival chances. As such, schistosomes use the heat shock protein (Hsp) machinery to escape the various insults associated with their life cycle [118]. Hsps form part of the cell’s response to stress; they are conserved and ubiquitous molecules that facilitate folding of other proteins (substrates/clients) during cellular stress [119]. Various heat shock response-related genes, including Hsp40 [120], Hsp90 [121], Hsp70 [122,123] and Hsp60 [124], have been identified in schistosomes. The most extensively studied group of stress proteins in schistosomes belongs to the Hsp70 family [118]. The remarkable conservation of its primary structure and its expression in a number of organisms make it an interesting target for diagnosis and treatment in various diseases. The therapeutic potential of Hsp70 in schistosomiasis has been explored in the literature [35,122,125]. Although Hsp70 is regarded as a potential drug target, its expression is only pronounced in the cercaria-shistosomula stage of the parasite. Studies reveal that treatment of cercariae with an Hsp70 inhibitor results in a change in the swimming behavior of the worm, reducing the chances of successful penetration of the host [126]. In contrast, Hsp60 is reported to be constitutively expressed throughout the entire life cycle of the worm, where it plays a major role in import and protein folding in the transition from cercariae to schistosomula [127] and is proposed to play a role in the survival of the schistosome under hostile conditions, thus making Hsp60 an interesting target for the treatment of schistosomiasis.

### 5.1. S. mansoni Hsp60

The 60 kDa Hsp is a specialized but ubiquitous group of molecular chaperones, generally known to form large oligomeric rings and function in folding of nascent polypeptides transported to the mitochondria with the assistance of the co-chaperonin, Hsp10, in an adenosine triphosphate (ATP)-dependent manner. The mechanism by which this protein allows for the survival of the worm has not yet been elucidated. We, therefore, propose, based on the mechanism of action of the human Hsp60, that after the exposure of the worm to the defense mechanism of the snail host, changes in temperature or the immune response of the human host upon penetration induces increased expression of *S. mansoni* Hsp60. Temperature variations have been suggested to influence the complex interactions that occur between schistosomes and their intermediate snail host [128]. A number of studies revealed that schistosomes have reduced tolerance to low temperatures, while their intermediate snail hosts show better tolerance to lower temperatures [129,130]. It has been reported that the rate at which miracidia mature to cercariae within the snail is relatively slow during cold weather conditions and cercarial shedding is subsequently reduced. Additionally, low *S. mansoni* infection rates have been reported for snails kept at lower temperatures (23 to 25 °C) in comparison to those kept at higher temperatures of 26 to 28 °C [128]. A rise in temperature from 26 °C to 32 °C has been reported to result in an increased shedding of *S. mansoni* cercariae; although these temperature conditions favor transmission of infection, they are detrimental to the snail host [131]. Mangal et al. [130] showed that constant high temperatures (>35 °C) result in low cercarial production, high snail mortality and eventually cause changes in the dynamics of the disease from stable endemic infection to unstable infection. Similarly, Adekiya and colleagues [128] also reported that temperatures above the optimal (26 to 32 °C) result in a decrease in the infection rate of the parasite due to the low abundance in snail production, thus limiting the growth and development of the parasite.

**Figure 2:**
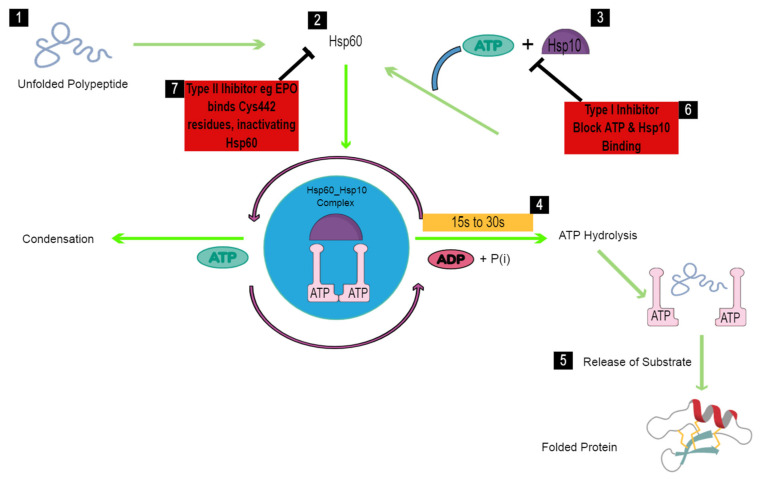
Schematic diagram of the folding mechanism of the schistosomal Hsp60-Hsp10 complex. In the human host, schistosomes are exposed to extreme temperatures that result in unfolding of their proteins (1). Increased temperature conditions within the worm stimulate the expression of Hsp60, which binds the unfolded polypeptide (2). Hsp10 and ATP immediately bind to Hsp60, forming an Hsp60-Hsp10 complex that facilitates folding in an ATP-dependent manner (3). Protein folding is, therefore, expected to occur within 15–30 s prior to ATP hydrolysis (4), which leads to the release of the bound polypeptide (5), and thus, the worm regains functionality and survives. To block the chaperoning activity of Hsp60 in schistosomes, a type I Hsp60 inhibitor such as Mizoribine binds to Hsp60, blocking ATP and Hsp10 binding, and thus, preventing protein folding (6). A type II Hsp60 inhibitor, Epolactaene, binds the Cys442 residue, thus inactivating Hsp60 prior to binding of the unfolded polypeptide (7).

As previously mentioned, these hostile conditions lead to abnormal changes in the proteome of the worm, such as unfolding of proteins. In the absence of defense mechanisms of the worm, the loss of the three-dimensional structure of proteins within the worm, accumulation and aggregation of unfolded proteins would result in reduced cell viability and tissue dysfunction. This will ultimately reduce the ability of the worm to perform its function and eventually lead to its death. In the presence of *S. mansoni* Hsp60, unfolded polypeptides exposing hydrophobic amino acid residues bind to the apical domain of *S. mansoni* Hsp60 that is not yet occupied by its co-chaperonin *S. mansoni* Hsp10 [132]. Subsequently, Hsp10 and ATP bind to the *S. mansoni* Hsp60 polypeptide complex, forming a *cis* ring complex. Binding of *S. mansoni* Hsp10 results in the burial of the hydrophobic polypeptide recognition regions of the apical domain of *S. mansoni* Hsp60, allowing for the subsequent release of the bound non-native substrate into the central cavity [133]. The non-native substrate is encapsulated in a comparatively polar environment, which supports folding and the formation of the native polypeptide state [134]. Folding of the non-native substrate is controlled by ATP, in the sense that the substrate is allocated about 15 to 30 s to fold prior to ATP hydrolysis [135]. Hydrolysis of ATP triggers the dissociation of *S. mansoni* Hsp10 from *S. mansoni* Hsp60, opening the folding cage and allowing for the subsequent release of the native form of the polypeptide (Figure 2). Generally, substrate proteins undergo repeated rounds of folding before the attainment of the native state; hence, substrates that were not properly folded are rapidly captured and undergo a second round of assisted folding. In this way, the parasite is able to escape the hostile conditions associated with its life cycle and to develop into adult worms that produce eggs that re-start the infection cycle. Owing to the important role played by *S. mansoni* Hsp60, the development of specific inhibitors that target this protein and can modulate its function in schistosomes can be an interesting approach in the design and development of anti-schistosomal drugs.

### 5.2. Hsp60 Inhibitors

Although a number of inhibitors have been developed for various Hsps, very few have been developed for Hsp60. Known Hsp60 inhibitors are from either natural or synthetic compounds and are grouped into two types [43,136]. Type I inhibitors block ATP binding and hydrolysis, subsequently blocking the Hsp60/10 refolding machinery because the ATP-dependent conformational changes are affected. Type II inhibitors are compounds that bind to the cysteine residues of Hsp60; however, details of their binding sites and mechanism of action are not yet understood. Natural compounds known to inhibit Hsp60 are mizoribine, epolactaene and myrtucommulone, while *o*-carboranylphenoxyacetanilide 8 and gold (III) porphyrin are Hsp60 inhibitors from synthetic sources [137]. To date, there have been no reports on the structure of schistosomal Hsp60; hence, inhibitors targeting the schistosomal homolog have not yet been identified.

## 6. Current Status of Schistosomiasis Treatment

The drive to control as well as eliminate schistosomiasis continues, with various strategies being put in place. However, despite all the efforts made, there is still a gradual rise in the number of schistosomal infections. To date, no new drug has been approved as an alternative to PZQ [138]. The WHO recommends the use of PZQ for the treatment of schistosomiasis in view of its effectiveness against all three *Schistosoma* species known to infect humans [3]. However, the drawbacks associated with its use necessitate improved and affordable treatment methods, including targeting protein interactions observed during the course of schistosomal infection.

Although PZQ remains the only accepted and available treatment for schistosomiasis, other drugs have been introduced but have faced limited use. Combination treatment of PZQ with one of the drugs that have been taken off the market, artemether, has been reported to have higher efficacy levels. Artemisinins are derived from extracts of sweet wormwood (*Artemisia annua*), a plant endemic to China, and are well established for their antimalarial activity. Their efficacy also extends to related parasitic infections, as they have been found to possess anti-schistosomal properties [139]. In support of this, a derivative of artemisinins, artemether, has effectively controlled *S. mansoni* and *S. japonicum* infections in areas of high endemicity such as in China [11]. In contrast to PZQ, derivatives of artemisinins, such as artemether, are more effective against immature schistosomes and show reduced efficacy against the invasive adult stages [140]. Although artemether alone is associated with lower cure rates than PZQ, results from clinical trials show that a combination of artemether and PZQ is more effective than PZQ alone [14]. Therefore, joint treatment would target all the developmental stages of the parasite in its definitive host and consequently prevent re-infection. A limitation to the use of artemisinin derivatives in the treatment of schistosomiasis is the risk of the development of resistant plasmodia, especially in sub-Saharan Africa, where schistosomes and *Plasmodium* species co-exist.

Previous studies have put forward a notion that in conjunction to improvements that can be made in terms of adherence to treatment and ensuring wide access to available treatment, the development of potential vaccine candidates offers the best option to increase the possibility of achieving WHO goals of eliminating this disease [141]. Although the current drug has been effective to some extent, there is general agreement among experts that sustainable reduction in the transmission and disease burden of schistosomiasis can only be achieved through vaccination. An effective anti-schistosome vaccine would greatly contribute to reduced schistosomiasis-associated morbidity through protective immune responses, leading to reduced worm burdens and decreased egg production [142,143]. There is currently no schistosomiasis vaccine, although a number of candidates are in various stages of clinical trials (Table 3).

### 6.1. Tetraspanin Proteins

Tetraspanins (TSP) are four transmembrane domain proteins that are found exposed on the surfaces of all multicellular eukaryotes [144]. These proteins are often localized at the plasma membrane and they interact with one another, as well as with immunoglobulin superfamily proteins, proteases and receptors. Through these interactions, they mediate various functions, including the regulation of cell signaling, cell proliferation, adhesion, spreading, migration, cell-cell fusion and pathogen entry of a number of diseases [145]. In schistosomes, TSPs are abundant in both the larval and adult stages of the parasite and while the exact functions of schistosome tetraspanins are unknown, their mammalian homologs are associated with partner proteins in the basolateral domains of the plasma membrane and function in cell–cell interactions and maintenance of cell membrane integrity. It is, therefore, believed that schistosome tetraspanins might perform a similar role in the tegument [146]. The failure to develop an antischistosomicide with high efficacy can be attributed partly to the complex immune-evasive strategies employed by the parasite to avoid elimination from its intravascular environment. Schistosomes have a unique outer syncytial surface, the tegument, which constitutes the host–parasite interface, and thus, represents an interesting tissue to target for the development of new control strategies. This can be achieved by identifying proteins that contain membrane-targeting signals that are expressed in the tegument.

The major TSP proteins in *S. mansoni* are Sm-TSP-1 and Sm-TSP-2. The full-length Sm-TSP-2 is a protein proposed to play a role in both tegument creation and maturation in the adult worm [2,149]. To determine the potential of these proteins as vaccine candidates, a study was conducted involving the vaccination of mice with recombinant Sm-TSP (rSm-TSP) proteins, which resulted in reductions of 57%, 65% and 64% in rSm-TSP-2 and 34%, 69% and 52% in rSm-TSP-1 for mean adult worm burdens, fecal egg counts and liver egg burdens, respectively. Fecal egg counts were drastically reduced by 65% and 69%, respectively, in both test groups. In particular, rS*m*-TSP-2 provided protection to mice over the 40% benchmark set by the WHO for the progression of schistosome vaccine antigens into clinical trials. Additionally, Sm-TSP-2 is strongly recognized by IgG1 and IgG3 antibodies from individuals that are naturally resistant to schistosomiasis, although exposed. This, in addition to the vaccine efficacy in mice, highlights the potential of Sm-TSP-2 as a safe and effective vaccine for human schistosomiasis [146]. It is postulated that tetraspanins in the tegument of schistosomula and adult worms may be receptors for host ligands, so vaccination with Sm-TSP-2 could induce antibodies that interfere with the interactions between these tetraspanins and their host ligands, thereby blocking crucial immune-evasive strategies by the parasite, thus rendering the schistosome surface vulnerable to an effective immune response [146,150].

The safety and immunogenicity of a candidate *Sm*-TSP vaccine has been assessed in a Phase I, double-blind, dose-escalation trial in which 72 healthy adults from a non-endemic area, aged 18–50 years, were randomized to receive three doses of recombinant *Sm*-Tetraspanin-2 vaccine formulated on aluminum hydroxide adjuvant (*Sm*-TSP-2/Al), with or without the adjuvant, as well as a glucopyranosyl lipid A aqueous formulation (GLA-AF), at intervals of approximately eight weeks between doses. Assessing clinical and serologic responses after the treatment, it was observed that anti-Sm-TSP-2 IgG responses were noticeably lower among recipients of Sm-TSP-2/Al without GLA-AF, while the recipients of Sm-TSP-2/Al with GLA-AF showed significant increases in IgG to Sm-TSP-2 [139]. It can, then, be concluded from this study that Sm-TSP-2/Al with or without GLA-AF is safe and well tolerated, although minor side effects such as headaches and pain at the injection site have been reported. In the United States and Brazil, Phase I clinical trials for the Sm-TSP-2/Alhydrogel^®^ schistosomiasis vaccine continue to determine the toxicity, safety and immunogenicity of this vaccine in healthy adults [148].

### 6.2. Paramyosin

Paramyosin is a myofibrillar 97 kDa protein anchored in the muscle layers and the tegument of schistosomes that has for many years been regarded as a potential vaccine candidate against *S. japonicum* (Sj97) and *S. mansoni* infection associated with resistance to infection and re-infection [2]. An earlier study showed that mice vaccinated with purified paramyosin free of an adjuvant resulted in 62–86% resistance against *S. japonicum* infection [151]. In addition to preventing infection, a longitudinal treatment re-infection study conducted in the Philippines, with Sj97 showing a longer time for humans to become re-infected and even lower re-infection intensity after treatment with PZQ [152,153]. Furthermore, it was reported that individuals who produce IgE but not IgG4 in response to rSj97 have a 77% lower re-infection chance after 12 months or more of treatment with PZQ [152]. Sj97 is now undergoing early preclinical testing to assess its efficacy and safety [142,149,152]. In the case of *S. mansoni*, a study to determine the efficacy of S*m*97 antigen in immunized mice, found that S*m*97 induced 44.1%, 59.1% and 61% reduction in worm burden, intestinal egg loads and granuloma size, respectively, compared to the infected control group. Moreover, increased protective immunity was observed in mice and this was associated with high levels of specific anti-Sm97 IgG1 and IgG2 antibodies [147].

### 6.3. Calpain

Calpain is a calcium-activated neutral cysteine protease localized in the tegument of all developmental stages and inner tegument membrane of adult schistosome worms. It is a central antigenic protein in surface membrane biogenesis and renewal, which is a common mechanism frequently used by schistosomes to escape the harmful host immune response [148]. The inactivation of calpain has an inhibitory effect on the C3b component of complement and 5-hydroxytryptamine signaling pathways, bringing about the acceleration of surface membrane synthesis. The large subunit of calpain is called S*m*-p80 and is putatively expressed in all developmental stages of *S. mansoni* [143,154], making this antigen an excellent target for a schistosome vaccine because of its consistent immunogenicity, protective and anti-fecundity potentials, as well as its proposed important role in the immune evasion process [155]. Prime-boost vaccination (priming with DNA and boosting with recombinant protein) with Sm-p80, in combination with a resiquimod adjuvant, results in a 49% worm burden reduction, whereas 50% protection can be observed when using the recombinant protein (Sm-p80/GLA-SE) as primary and boost vaccine (immunized and then boosted by recombinant protein) in mice [156]. Following the same approach, but with a different adjuvant, oligodeoxynucleotide (ODN) 10104, a 70% worm burden reduction was reported with a 75% egg reduction, suggesting that ODN 10104 increases the efficacy of Sm-p80. The vaccine also elicits strong immune responses that include IgM, IgA and IgG isotypes [155]. This antigen has been tested in baboons (*Papio anubis*), where a considerable worm burden reduction of 58% was observed with the Sm-p80-based vaccine adjuvanted with resiquimod and CpG ODN [157]. Moreover, the vaccine provided levels of protection against *S. mansoni* infection in baboons comparable to those achieved by the irradiated cercarial vaccine. Antibodies and IFN-γ were shown to play a crucial role in the protective immunity observed in this non-human primate model [158]. In addition, it has been shown that Sm-p80 has a therapeutic effect in vaccinated baboons through a reduction in the numbers of established worms, reduced retention of eggs in tissues and decreased number of eggs excreted in feces [155].

Interestingly, Sm-p80 has been shown to provide cross-species protection against *S. haematobium* in hamsters and baboons. A 64% reduction in tissue egg load and a 48% reduction in worm burden were observed in hamsters vaccinated with recombinant Sm-p80/GLA-SE. Similarly, in baboons, it resulted in a 64% egg load decrement in the urinary bladder and a 53% reduction in urine egg output [2]. A balanced pro-inflammatory (TH17 and TH1) and TH2 type response consequently occurred after vaccination, indicating that the Sm-p80 vaccine is effective against both intestinal and urinary schistosomiasis and will, thus, be significantly advantageous in reducing the overall burden of schistosomiasis [154]. The recombinant Sm-p80/GLA-SE vaccine, “SchistoShield,” is expected to move forward to Phase I and II human clinical trials [97,149,159].

### 6.4. S. mansoni 29 Kilodalton Protein

Specific antibodies against Sm29, IgG1 and IgG3 are detected in sera of patients living in endemic areas of Brazil and significantly higher levels of these antibodies were found in individuals who are resistant to re-infection [105]. Mice immunization with rS*m*29 has been shown to result in a significant reduction in adult worm burdens, liver granuloma counts and intestinal eggs, as previously stated. In addition, three doses of rSm29 are enough to elicit significant host protection levels, ranging from 26% to 48%, to induce significant production of IL-2, IFN-γ, IL-17 and IL-4, as well as specific antibodies such as IgG, IgG1, IgG3 and IgE, and an increase in the percentage of CD4+ central and effector memory cells. It has been proposed that rSm29 is capable of inducing protection in previously infected animals, reinforcing its potential as a vaccine candidate [160]. Immunizing mice with Sm29 vaccine preparation containing alum as an adjuvant (Sm29Alum) has been shown to render protection against re-infection at levels between 29% and 37%. On the contrary, immunization with the Mono-phosphoryl Lipid A adjuvant (Sm29MPLA) does not provide any protection against reinfection. In particular, immunizing mice with Sm29Alum formulation resulted in higher production of IgG, IgG1 and IgE following all immunization doses. Therefore, it can be concluded that Sm29Alum effectively protects against *S. mansoni* reinfection in mice [161].

Proteins usually form complexes to elicit a specific function. Examples of these include Sm29 and Sm14, which form a fusion protein designated FSm14/29. The efficacy of the fusion protein has been tested in mice where Polyinosinic-poly cytidylic acid [poly (I:C)] adjuvanted and unadjuvanted FSm14/29 vaccinated mice showed a significant reduction in adult worm burdens of 48.4% and 44.7%, respectively. In addition, significant reductions of 82.8% and 73.5%, respectively, were observed in liver egg burden. Likewise, the intestinal egg count reduction reached 72.8% and 76.6% for the adjuvanted and unadjuvanted FSm14/29 antigen, respectively. Finally, adult worms recovered from immunized mice displayed deleterious structural changes. Another study showed that fusing Sm29 with *Sm*-TSP-2 results in increased worm burden reductions from 22% to 35%, a higher titer of IgG1 and IgG2 antibodies, as well as improved levels of IFNγ and TNF-α than when Sm29 is used alone in challenged mice [149,162]. It is clear that the adjuvant greatly improves host protection and combines the concept of using multi-antigen fusion proteins as vaccine candidates against *S. mansoni* [116].

### 6.5. Sm14

Sm14 has long been considered a potential vaccine candidate because of its critical biological function in facilitating absorbance, transport and compartmentalization of fatty acids from the host [163]. Recombinant Sm14 (rSm14) was tested in mice for host protection and it was shown that it provides up to 67% protection in terms of a reduced *S. mansoni* worm burden in mice without the use of an adjuvant. Reassuringly, no autoimmune response was observed even though its structure is identical to the basic form of mammalian host homologues [115]. Moreover, it has been shown to be cross-species protective against both *S. mansoni* and *Fasciola hepatica* infection. The development of a dual vaccine effective against both fluke infections will be a great milestone in terms of human and animal health. Recombinant Sm14 with glucopyranosyl lipid adjuvant stable emulsion (GLA-SE) adjuvant has entered and successfully completed Phase I clinical trials in healthy adult volunteers in Brazil, authorizing its status as safe and immunogenic [164].

The Phase I clinical trial of adjuvanted rSm14 was conducted on 20 male volunteers from a schistosomiasis non-endemic area in Brazil. Thirty days after the first vaccination, significant increases in Sm14-specific total IgG, IgG1 and IgG3 were observed, and in IgG2 and IgG4 after 60 days. Additionally, 88% of vaccinated subjects were shown to produce high titers of Sm14-specific IgG antibody 90 days after the first vaccination injection. A complex mixture of Th1 and Th2 cytokines are released from peripheral blood mononuclear cells after rSm-14 stimulation. The Th1 response is characterized by IFN and TNF; these cytokines are responsible for controlling the effects of parasite eggs on the liver, a situation that is rapidly followed by a strong egg-induced Th2 response (IL-5, IL-10), which prevents severe consequences and protects the liver by downregulating the inflammatory mediators. The rSm14/GLA-SE vaccine presents itself as a strongly immunogenic and safe vaccine [164]. Further immunogenicity and safety Phase II trials of rSm14/GLA-SE are intended for schistosomiasis-endemic areas in Brazil and Africa [165].

### 6.6. Schistosoma mansoni Kunitz Type Protease Inhibitor

The Kunitz inhibitor is a 16 kDa Kunitz-type protease inhibitor (KI-1) actively present in the excretory-secretory products, tegument of adult worms and the sub-shell region of eggs. *Schistosoma mansoni* KI-1 inhibits numerous proteases, which possess anti-inflammatory and anti-coagulant properties, in this way supporting parasite survival in an extremely hostile environment and perhaps playing a critical role in host immune modulation. Secretion of SmKI-1 by schistosome eggs was previously explained as important for their migration through the intestinal wall and into the gut lumen for excretion in human stool to the external environment. To confirm the effect of this inhibitor, rSmKI-1 vaccine trials were performed in mice and a reduction of 23–33% in adult worms, 28–31% in intestinal eggs, 33–39% in fecal eggs and 20–43% in liver eggs was observed. Moreover, rSmKI-1 increased the production of IFN-γ, IL-10 and IL-6 to a considerable level in vaccinated mice, maintaining a Th1/Th2 type balanced response. Recombinant SmKI-1 resulted in partial protection against *S. mansoni* in the murine model of infection and could be established as part of a combination vaccine with other vaccine candidates to provide a more solid level of protection [71,166].

### 6.7. Schistosoma mansoni Asparaginyl Endopeptidase (SmAE/Sm32)

S*m*32 is a cysteine protease of the legumain family [167] that is released as an excretory–secretory material, where it is actively involved in the hydrolysis of pro-proteins that are involved in the degradation of hemoglobin expressed in the gastro-dermal cells of the schistosome gut and head glands of the cercariae [148]. Moreover, S*m*32 has been found to be the principal source of amino acids for the parasite. This antigen induces an antibody-mediated inhibition of its catalytic activity, in this way preventing the processing of cathepsins such as B and L, which are actively involved in hemoglobin digestion, making it an interesting potential vaccine target for schistosomiasis. The inhibition of the above-mentioned activities has been shown to result in intense reduction in the egg burden of adult worms [168]. Additionally, the immunolocation of Sm32 in the head glands of cercariae [169] suggests a possible function of this protease in host tissue invasion. Therefore, Sm32 could be considered an interesting target, as it affects the nutrition of adult worms, thereby reducing worm burden, viability and fecundity, while simultaneously interfering with host penetration by cercariae [170].

## 7. Future Prospects of Protein Vaccine Candidates against *Schistosoma* Infection

Vaccine development is a long process that can take decades. To date, three schistosome protein vaccine candidates that have been produced by good manufacturing procedures have entered safety and immunogenicity human clinical trials. These are Sm14 [171], Sm-TSP-2 [148] and Sm-p80 [159]. Phase I clinical trials of rSm14/GLA-SE products indicated that they were safe and strongly immunogenic against schistosomiasis in healthy individuals. The next phase of testing is expected to take place in schistosomiasis-endemic areas of Brazil and Africa [164,171]. The Sabin Vaccine Institute product development partnership is focusing on the development of the Sm-TSP-2/Alhydrogel^®^ schistosomiasis vaccine. This vaccine entered Phase I in 2017 to determine immunogenicity, reactogenicity and safety in healthy, non-infected adults in the United States and Brazil [148]. Moreover, the recombinant Sm-p80/GLA-SE vaccine, “SchistoShield^®^,” has entered Phase I and II clinical trials [149]. Other antigen candidates that are in pre-clinical trials at various stages include S*m*97, Sm29 and SmKI-1 for *S. mansoni* infection [4].

Gene sequencing of schistosome species and modern proteomics methods have significantly promoted the discovery of many novel possible vaccine and drug targets, as well as diagnostic biomarkers, via high-throughput and sensitive proteomics methods. In particular, immuno-proteomics approaches are essential in screening proteins from different schistosome species in both animal models and clinical sources. Moreover, protein microarray and microplate immuno-proteomics allow for unlimited virtual screening of any antigen. This includes surface-exposed hypothetical proteins from the predicted proteomes of schistosome species [172]. The advancement of new technologies, including genomics, transcriptomics, microarrays and immunomic profiling, has helped significantly in the identification of promising new schistosome target proteins, which are crucial for the survival of the parasite in the host [172].

The combination of two different proteins that interact to perform a similar function has been shown to result in higher levels of vaccine-induced protection. Targeting key biological functions of schistosomes that are critical for the survival of the parasite, such as nutrient uptake, metabolism and tegumental integrity, represent key potential sites to consider for the elimination of the schistosome through vaccination and/or chemotherapy. Although significant progress has been made in previous years in the identification of promising vaccine/drug candidates, schistosomiasis drug/vaccine development has proven highly challenging and costly; hence, many vaccine and drug candidates fail to even make it to clinical trials [149].

## 8. Conclusions

Schistosomiasis is most prevalent in resource-poor areas where farming and fishing activities are common to sustain households. As such, pharmaceutical industries do not put much effort into developing various treatment options for this disease, as it mostly affects people who cannot afford highly priced drugs and do not have access to good health care systems. For these reasons, this disease falls under the ‘neglected diseases’ category. It has been reported that this ailment predisposes its sufferers to bladder cancer, human papilloma virus and HIV, which all cause a high disease burden in many countries, especially in sub-Saharan Africa. This has raised concerns about a search for alternative treatment options to supplement PZQ. Targeting specific protein interactions within the schistosome is an interesting approach owing to the growing interest in the therapeutic potential of protein modulators. Although no drug has been designed by targeting PPIs in schistosomiasis, a few protein interactions have shown potential as therapeutic targets against schistosomiasis, such as *S*m29 and *S*m14. In addition, targeting protein interactions that have the potential to assist schistosomes in acquiring resistance against PZQ and escaping the immune defense mechanism of the host will lay a basis for anti-schistosomal drug discovery through PPIs. The oxidative stress response by the host immune defense system and incubation of schistosome worms with sub-lethal doses of PZQ lead to an increase in oxidative stress in the worm, resulting in cellular protein unfolding and aggregation in the endoplasmic reticulum, causing loss of cell viability and function in the worm. This induces the upregulation of a family of chaperone proteins, specifically the Hsp60 and Hsp10 chaperonin complex, which assists the worm to overcome the oxidative stress response and decreases its sensitivity to PZQ. An exploration of the actual mechanism by which this protein complex allows the worm to escape host immune responses will lay the ground for determination of specific protein inhibitors that can potentially modulate its function in vitro and in vivo. Inhibitors of this protein complex will not only assist in the development of new drugs, but can potentially reduce the resistance levels of schistosomes to PZQ.

The infection process in schistosomes is facilitated by the phototaxic cercariae, which move towards the surface of shallow water, where they maximize their chance of contact with humans. The cercariae follow a thermal gradient to find their potential hosts and when they make contact with human skin, they release chemical signals, including medium-chain fatty acids such as linoleic acid, to stimulate skin invasion. The first step in the invasion process is the release of gland contents from the acetabular gland complex of the posterior of the cercarial head, mainly SmCE, which facilitates invasion by degrading the dermal elastin. It is, therefore, clear that SmCE plays a significant role in the initiation of infection. The identification and characterization of SmCE-specific inhibitors such as Z-AAPF-CMK and Elafin, thus, present an interesting approach to reducing schistosomal infection. On a practical level, it has been suggested that studies on protease inhibitors can advance the understanding of host–parasite interactions and lead to the identification of novel vaccine candidates and/or drug targets against schistosomes [71].

For drug design purposes, it is evident that neutrophils and other effector cells elicit an immune response using elastases to fight parasites; this presents an opportunity to study specific inhibitors that can break the interaction of Smpi56 with these elastases, specifically NE and pancreatic elastase. Current control efforts rely on antihelminthic treatment, but to sustain their effects, drugs must be applied repeatedly and for an indefinite period, which is costly. Moreover, reports of increased rates of re-infection after mass treatment limit strategies based on chemotherapy alone. As such, a prophylactic vaccine seems the ideal method for sustainable control of schistosomiasis, alone or in combination with the current drug. Although the vaccine development process is known to be long and costly, currently, three promising vaccine candidates for *S. mansoni* (Sm14, SmTSP-2 and Sm-p80) are in different stages of clinical trials. Other candidates, including Sm97, Sj97, Sm29 and SmKI-1, are also in various stages of pre-clinical trials. It is hoped that new lines of inquiry building on the findings detailed in this review will advance the understanding of host–parasite interactions and lead to the recognition of new drug targets and vaccine candidates against the schistosome parasite through specific interactions between host and parasite proteins.

## Figures and Tables

**Table 1:** Uncharacterized Schistosome serpins.

*Schistosoma mansoni*					
Database Gene Name	Size (kDa)	Reactive Central Loop Amino Acid Sequence	Potentially Targeted Protease	Similarity to Mammalian Serpin	Reference
Smp_003300	43.7	EDGVEAAAATVMGIGLRSA	SmCl2	α1-antitrypsin (53%)	[53]
Smp_090080	46.0	ESGIEATTVTSPIFVPISA	Elastase-2	Neuroserpin (48%)	[54]
Smp_090090	46.3	EVGMEARSVANAMFIPLSS	CT, Cat	Neuroserpin (52%)	[50]
Smp_062080	43.3	EQGVVAAAASSVEVVQLSA	Cercarial elastase	SCCA-2 (53%)	[55]
Smp_155530	51.0	EEGVVAAGVTACVFDNCDS	Peptidase complex (SPC)	SCCA-2 (55%)	[50]
Smp_155550	43.5	EKGAVAAAATATRMIRCTA	Peptidase complex (SPC)	PAI-2 (53%)	[50]
Smp_155560	43.6	EKGAVAAAATATQMVRCTA	Peptidase complex (SPC)	SCCA2/SCCA1 (52%)	[56]
Smp_155560	43.8	EKGAVAAAATATQMVRYSA	Peptidase complex (SPC)	PAI-2 (54%)	[56]
***Schistosoma japonicum***					
Sjp_0113720	26.4	EKGVEAAAATAIYSLGRSL	Thrombin	Ovalbumin (52%)	[50]
Sjp_0076600	45.0	EEGAVAAAASATVMLKCSA	Peptidase complex (SPC)	SerpinB6c (57%)	[50]
Sjp_0085750	45.6	ESGIEAASVTSPIIVPISA	Elastase-2	Neuroserpin (50%)	[50]
Sjp_0113080	43.0	EKGAEAAAATATKIIPLSL	Cercarial elastase	Proteinase inhibitor 6 (53%)	[50]
***Schistosoma haematobium***					
ShSPI	45.9	ESGIEATTVTSPIFVPFSA	Cercarial elastase	Ovalbumin (48%)	[57]

**Table 2:** Schistosome protease inhibitors.

Protease Inhibitor	Specie	Protease Inhibited	Location of Expression	References
SmSrpQ	*Schistosoma mansoni*	SmCE	Cercariae	[74]
SmPi56	*Schistosoma mansoni*	NE	Adult worms	[50,71,73]
ShSPI	*Schistosoma haematobium*	Thrombin	Surface of adult worms	[89,98]
SjB10	*Schistosoma japonicum*	Trypsin, chymotrypsin, pancreatic elastase	Cercariae, schistosomula, eggs, adult male worms	[97]
SjB6	*Schistosoma japonicum*	Trypsin	Eggs	[89]
SmKI-1	*Schistosoma mansoni*	Trypsin, chymotrypsin, NE	Adults, schistosomula, eggs	[67,71]
SjKI-1	*Schistosoma japonicum*	Trypsin, chymotrypsin, NE	Eggs, adult worms	[71]
SmSPI	*Schistosoma mansoni*	Chymotrypsin, PE, NE	Head gland of schistosomules, spines of adults	[57]

**Table 3:** Anti-schistosomal vaccine candidates.

Antigen	Location	Target Species	Function	Vaccine Development Stage	References
*Sm*-TSP-2	Tegument	*S. mansoni*	Maintains tegument creation and maturation in the adult worm	Phase I	[146]
S*j*97	Tegument of schistosomula and penetration glands of the cercariae	*S. japonicum*	Associated with resistance to infection and re-infection	Pre-clinical	[2]
S*m*97	Tegument and cercariae	*S. mansoni*	Offers protective immunity to the host	Pre-clinical	[147]
S*m*-p80	All developmental stages of *S. mansoni*	*S. mansoni*	Immune evasion	Pre-clinical	[148]
*Sm*29	Tegument	*S. mansoni*	Protection against re-infection	Pre-clinical	[116]
*Sm*KI-1	Tegument and sub-shell region of eggs	*S. mansoni*	Inhibits host proteases; permits parasite survival	Pre-clinical	[71]

## Data Availability

Not Applicable.
